# Planetary transmission performance tests at very low temperatures

**DOI:** 10.1038/s41598-022-26416-3

**Published:** 2022-12-17

**Authors:** Jakub Sikorski, Witold Pawlowski

**Affiliations:** Institute of Machine Tools and Production Engineering, Lodz University of Technology, Stefanowskiego 1/15, 90-537 Lodz, Poland

**Keywords:** Aerospace engineering, Mechanical engineering

## Abstract

This article presents the results of a study on resistance to motion in a multi-stage planetary transmission, built with lightweight structural materials such as aluminum alloy 2017, with bearing nodes featuring steel ball bearings made from X65Cr14 alloy and lubricated with molybdenum disulfide powder. Details of the planetary gear construction were presented, followed by operational performance tests. During the performance tests, the temperature of the running transmission was gradually lowered with liquid nitrogen to as low as − 190 °C. The analysis covered, among others, the power consumption of the mechanism as a function of temperature. The results were compared with the parameters of the mechanisms already working in space. The measurements were carried out to confirm the applicability of the gearing in drive systems of manipulators intended to operate in open space or under extraterrestrial conditions such as on Mars.

## Introduction

Planetary and strain wave gearsets have the most compact designs. In planetary transmissions, the torque transmitted by the gearing is nearly equally distributed over more than one gear wheel. Typically, their number ranges from 3 to 6, which enables high load capacity in spite of the compact size of the gearset. Additionally, these transmissions are usually designed for a gear ratio of 4–10, provide high stability and an efficiency of approximately 97%^1^. Even greater gear ratios, up to 5000, are offered by two-stage differential planetary transmission^2^, but the design of such a transmission is complicated.

**Figure 1:**
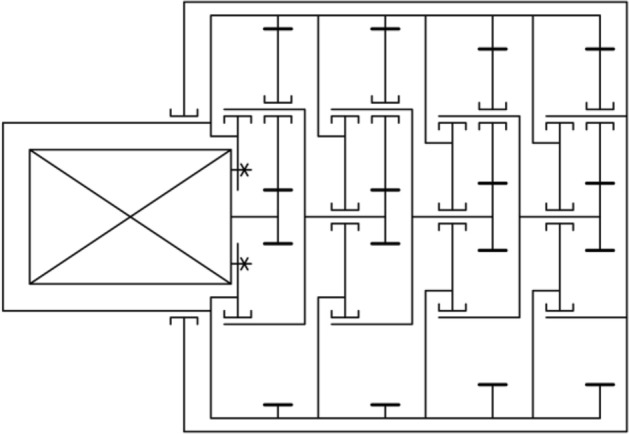
Diagram of the designed planetary gearing.

Planetary gearsets can be used in various configurations as reducers, multipliers, and differentials^1^. In the gearing assembly described in this paper, a reducer configuration was used with the ring wheel fixed, the sun gear positioned on the input shaft, and the pinion carrier connected to the output shaft. Figure 1 shows a diagram of the mechanism of the designed four-stage planetary gearset, where the pinion carrier of the ultimate stage of the gearset is connected to a part of the rotatable body.

The temperatures at which the transmission is anticipated to operate do not occur on Earth, thus when trying to find comparable designs, one has to look mainly to solutions applied in machines used on Mars. Temperatures on the surface of Mars range from − 140 to 27 °C due to the planet being 1.52 times further from the Sun than Earth is. That is why only 43% of the energy hitting Earth reaches an equivalent area on the surface of Mars^3,4^.

Given the conditions on Mars, the equipment used there, including gearsets, needs to withstand very low temperatures, which has been discussed in the studies on the design of Mars landers and rovers. The influence of temperature on the changes in the frictional moment during transmission operation is also very important^5^.

The Mars Volatiles and Climate Surveyor lander was fitted with a robotic arm, a manipulator with four degrees of freedom. Its actuators were able to generate torque of respectively: 26 Nm, 91 Nm, 53 Nm, and 10 Nm during normal operation, and 50% higher peak momentary torque. The actuators were designed as two-stage gearing containing a planetary gear and a harmonic gear or a planetary gear and a bevel gear. The gearsets were driven by DC brush motors. The overall ratios of the actuators were 4000 and 16,000. The mechanical systems of the actuators were designed to operate at temperatures from − 105 °C (− 90 °C) to 35 °C; to safeguard them against more extreme climatic conditions, the joints were fitted with 1 W and 4 W heaters^6–8^. The results of tests on the effect of temperature on the amperage required by the actuator motors during no-load operation showed that with decreasing temperature the power used by the actuator increased considerably.

The actuators were made from aluminum and titanium alloys. As for the construction designs of connecting members, the reports in the literature are inconsistent in that they refer to either carbon fiber composite or aluminum alloy as the material used^6–8^.

## Description of the transmission design

Autodesk Inventor Professional was used to design and analyze the gearset. It is parametric application software that facilitates 3-D part design. Virtual assembly of parts into subassemblies and complete machines is also possible.

**Figure 2:**
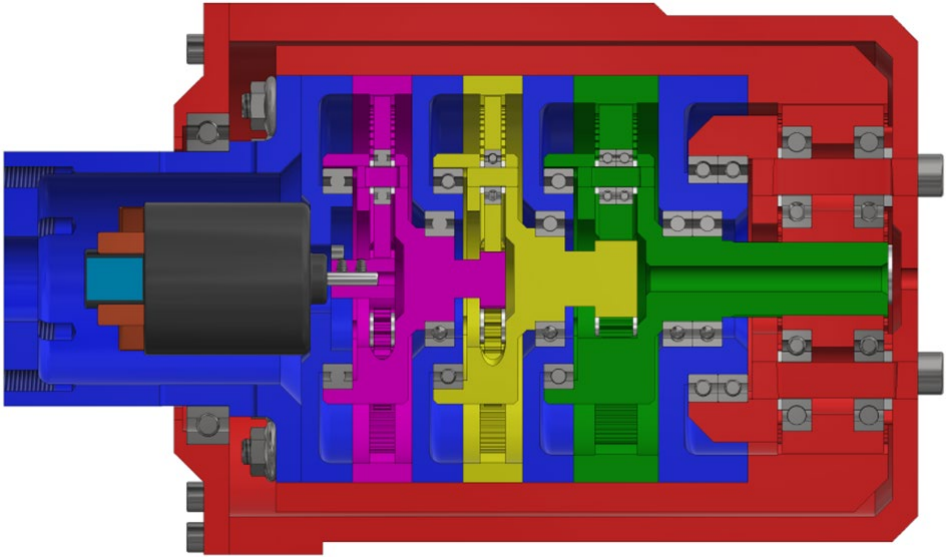
Section view of the gearset.

Based on the presented assumptions for operation at very low temperatures, the design of a four-stage planetary gearset driven by a brush electric motor was performed. A section view of the complete gearing is shown below in Fig. 2, where the individual colors denote:

- black: motor,
- grey: bearings and bolts,
- purple: first stage gearing,
- yellow: second stage gearing,
- green: third stage gearing,
- red: fourth stage gearing,
- blue: internal components of the body.

**Figure 3:**
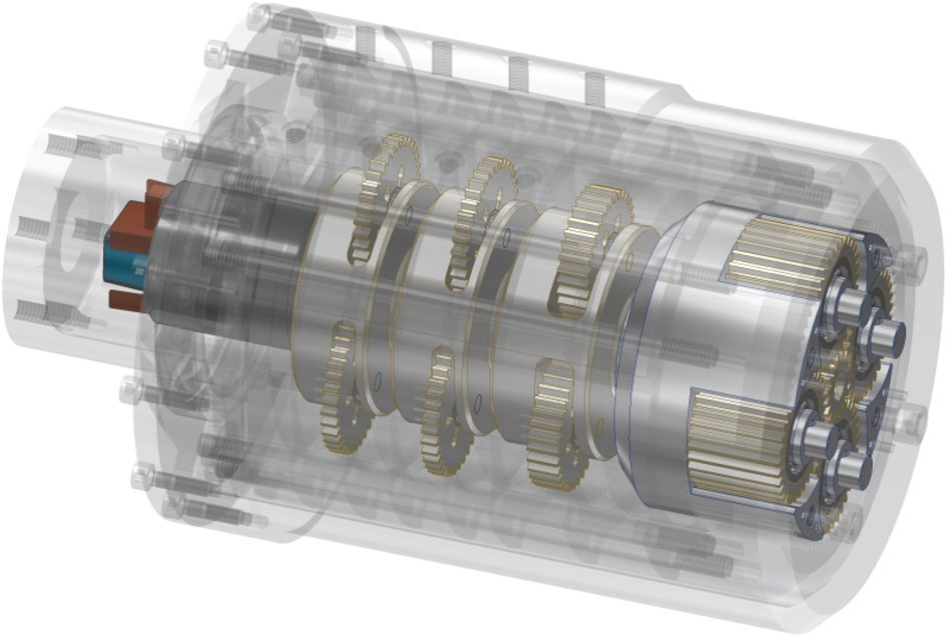
View of the interior of the transmission.

The transmission was powered by a 540 class brush electric motor, Absima Thrust B-SPEC 80 T, which has the idle speed of 5300 rpm when powered with a nominal voltage of 7.2 V, and the power output is 80 W. Figure 3 shows a view of the gearset interior with the motor, the planetary gears and carriers of the successive stages of the planetary gearbox highlighted.

**Figure 4:**
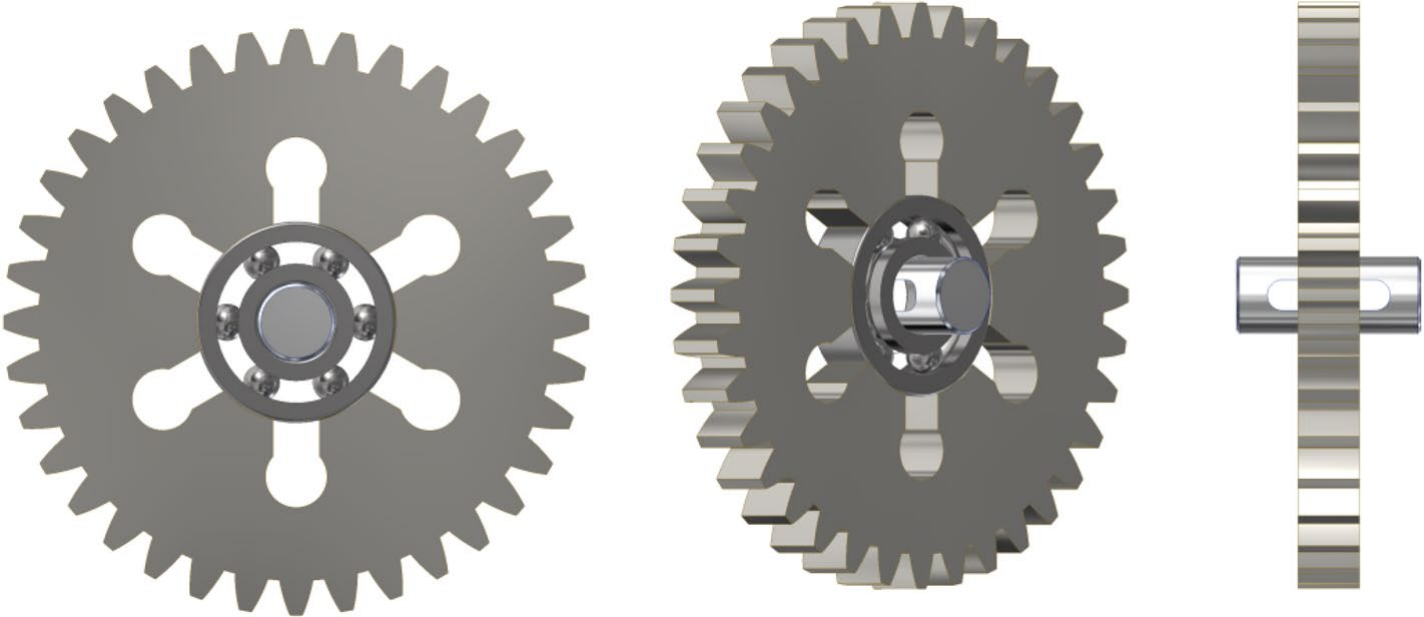
Views of the planet gear of the first stage gearset, with the bearing and pin.

To ensure reliable operation of the gearset across a broad range of temperatures, it was necessary to take a special approach to the design of the shape of the aluminum alloy gearset elements mating with the steel ball bearings. Accordingly, the gear wheels and pins, shown in Fig. 4, forming the bearing nodes have additional cutouts to enable dissipation of stresses caused by the difference in the coefficients of thermal expansion of these elements, equal to:

- aluminum: 23 * 10^–6^ K^−1^,
- steel: 12 * 10^−6^ K^−1^.

The gears of all transmission stages have a module of 1 mm, and the inter-tooth clearance has been set at 0.1 mm. This is quite a large value, but due to the prototype nature of the transmission, the maximum value of the clearance was chosen because the actual thermal deformations were not known, and the blocking of the transmission due to thermal expansion could lead to its premature failure. Additionally, the number of teeth of individual gears was selected so that the calculated total unit correction was 0.

Finally, based on the literature data, molybdenum disulfide powder was selected as a lubricant for the transmission mechanism. Comparative tests have shown that the application of this material results in the lowest resistance in the mating surfaces of the rolling bearing elements during operation at temperatures reaching − 190 °C, in an oxygen and moisture free atmosphere^9^.

Molybdenum disulfide is a true solid lubricant and does not require adsorption of additional substances to develop lubricating capacity^10^. The reason for this lies in the structure of this material containing alternating layers of sulfur and molybdenum, which during the abrasion of the successive layers release amorphous sulfur that forms lubricating film^11^. Since the compound performs best in an environment devoid of other substances, it can be used in vacuum and is the lubricant of choice for aerospace applications^11,12^.

Based on the limited literature data^13–16^, a comparison of the torque-to-mass ratio of the designed transmission with the existing designs was made. Data is presented in Table 1.

**Table 1:** A comparison of the torque-to-mass ratio of the designed transmission with the existing designs.

Actuator name	Operating temperature range	Mass (kg)	Torque (Nm)	Torque-to-mass ratio (Nm/kg)
**Mars exploration rover instrument deployment device**				
Azimuth	− 70 °C to + 45 °C	0.59	45	76
Elevation	− 70 °C to + 45 °C	0.48	45	94
Elbow	− 70 °C to + 45 °C	0.405	20	44
Wrist	− 70 °C to + 45 °C	0.38	9	24
Turret	− 70 °C to + 45 °C	0.35	9	26
**Mars science laboratory robotic arm**				
LPHTA	− 110 °C to + 50 °C	7.8	1143	147
WATER	− 110 °C to + 50 °C	4.24	259	61
**Designed transmission**				
	− 190 °C to + 30 °C	5.06	272	54

The above data indicate that the designed transmission, comparing it to the other mechanisms, has a comparable or slightly worse torque-to-mass ratio, but it is able to work at much lower temperatures, which allows to eliminate transmission heating systems, and thus reduce the total mass of the Mars rover.

## Description of the test bench

**Figure 5:**
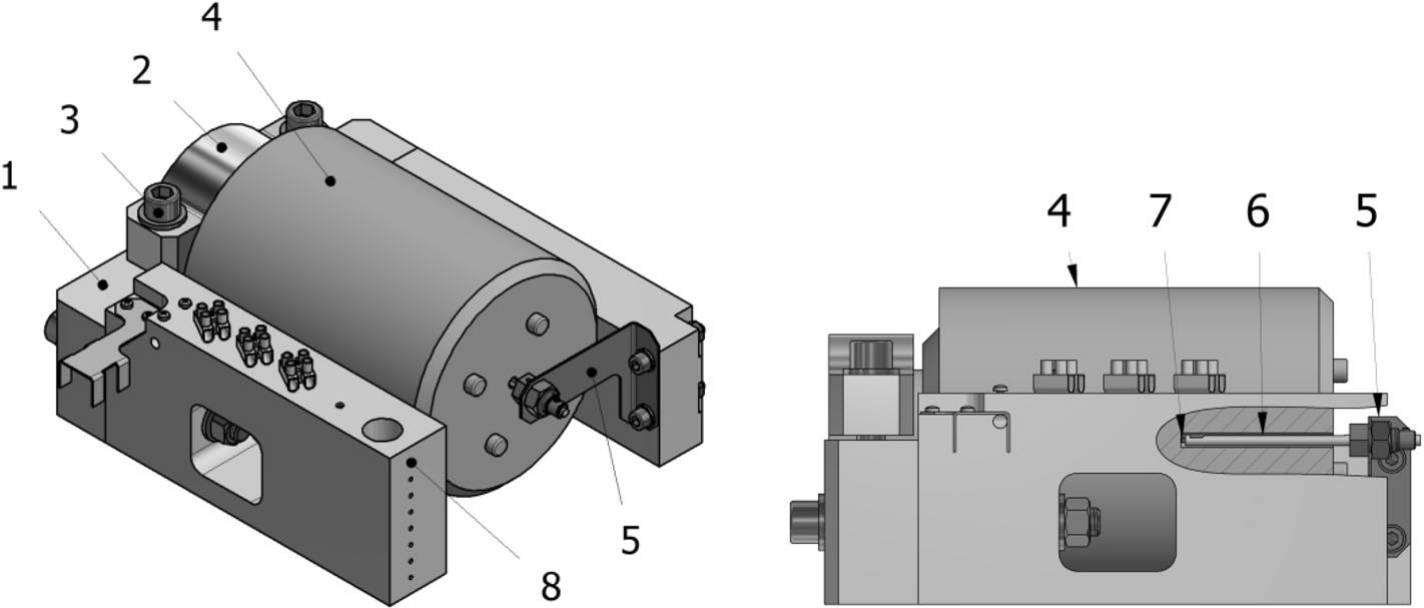
View and section of the gearset holder.

Before the transmission prototype could be tested, it first had to be secured within a custom-built, stable holder as shown in Fig. 5. The main components of the holder are supports (1) and a clamp (2) connected by bolts (3). The supports and the clamp, by virtue of their specific shape, hold the transmission motor housing (4). A probe bracket (5) positioned inside the transmission, at the end of which a temperature sensor (7) was mounted, was bolted to one of the supports. Additionally, at one end of the support, sensors (8) were installed to measure the temperature in the lower and upper parts of the interior of the thermal housing.

**Figure 6:**
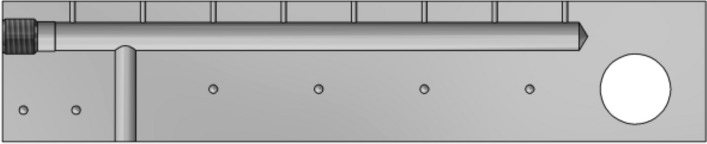
View of the liquid nitrogen distribution system.

A system of conduits was provided in one of the holder supports to enable even distribution of liquid nitrogen over the surface of the transmission, shown in Fig. 6. It was designed to ensure consistent cooling conditions over the entire surface of the transmission and uniform heat dissipation from the inside of the mechanism.

**Figure 7:**
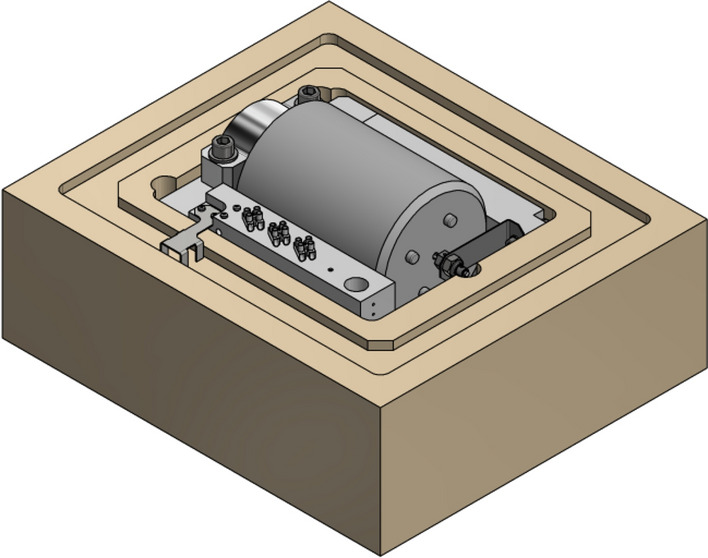
View of the transmission holder in the thermal casing.

The assembly was then placed in a two-part casing made of XPS polystyrene with a thermal transmittance of 0.035 W/(m*K), which ensured controlled thermal conditions. A view of the holder with the transmission located in the lower half of the thermal casing is shown in Fig. 7. Based on analytical formulas, taking into account the technical parameters of the thermal casing material, the dimensions of the interior and the wall thickness, the overall heat loss due to dissipation through the walls was calculated to be only 24 W. Unfortunately, the above value has not been experimentally verified because the liquid nitrogen stream entering the thermal casing and the temperature of the gas exiting the thermal casing were not measured.

**Figure 8:**
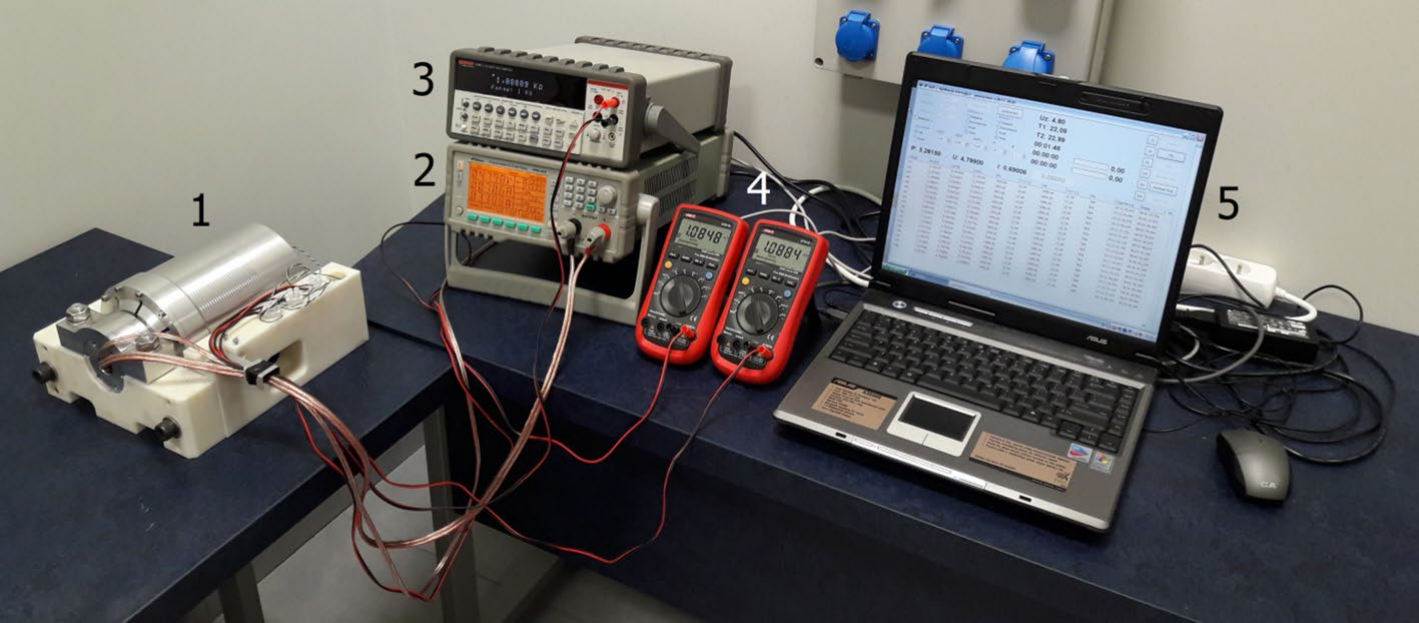
Transmission positioned in the holder and the measurement instruments.

The test bench is shown in Fig. 8. It included the transmission holder with a multi-stage planetary transmission (1). The electric motor used to drive the gearset was connected to a laboratory power supply unit (2). Other components comprised a laboratory meter (3) and universal meters (4) used for measuring the resistance of the temperature sensors integrated in the holder and inside the mechanism. Measurement data from the power supply unit and universal meters were recorded on a laptop computer (5) at a frequency of approximately 95 measurements per minute.

**Figure 9:**
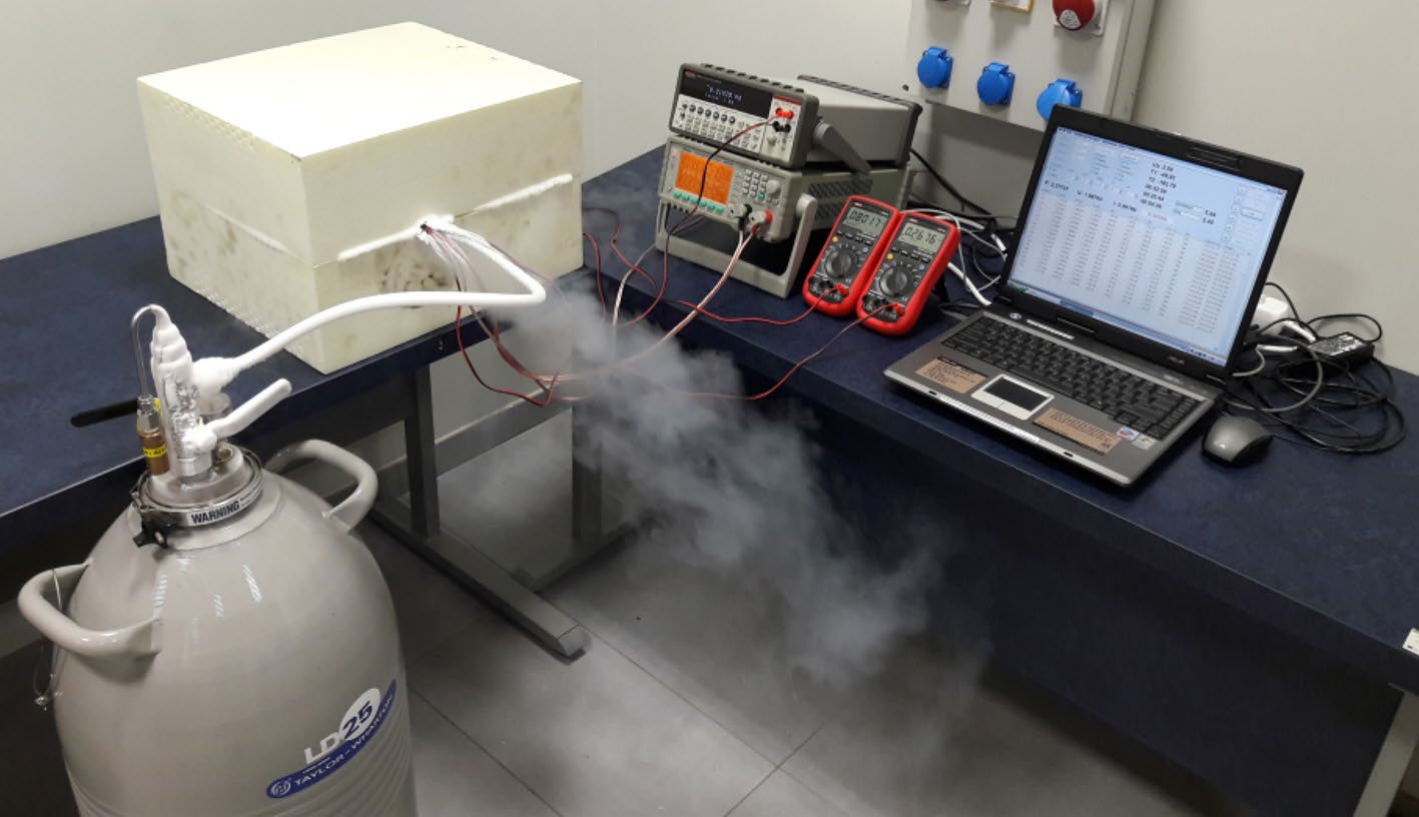
Test bench for measuring the power losses in the gearset operating under low temperatures.

Following initial tests at an ambient temperature, a low-temperature resistant Teflon tube was connected to the transmission holder, supplying liquid nitrogen from a Dewar tank, and the entire assembly was encased in a thermal casing as shown in Fig. 9.

## Research methodology

During the analysis of the operating characteristics of the gearset, the main parameter measured was the power required for the transmission to work. As a result, it was possible to determine the increase in the power required by the motor driving the transmission as a function of the decreasing temperature and to consider whether the entire mechanical system could operate at cryogenic temperatures. The tests provided a conclusively positive answer to the question whether appropriately designed structural elements would ensure that parts with different thermal expansion, of which the transmission was built, could work together.

The procedure to test performance of the transmission was conducted in two steps. First, the gearset worked at an ambient temperature. The results were then used as a starting point for the second step, in which the transmission operated at progressively lower temperatures down to as low as − 190 °C.

The measurements carried out at an ambient temperature showed that the gearing was able to operate smoothly over the entire range of the rotational speed of the motor, from 600 to 6100 rpm. The total operating time of the transmission during the tests was approximately 10 h. No disadvantageous phenomena were observed that would be a result of using only solid lubricant for the ball bearings and gear wheels. In addition, after the tests at ambient temperatures, the mating parts of the transmission were inspected. Traces of wear were detected on the teeth of the meshing gears.

**Figure 10:**
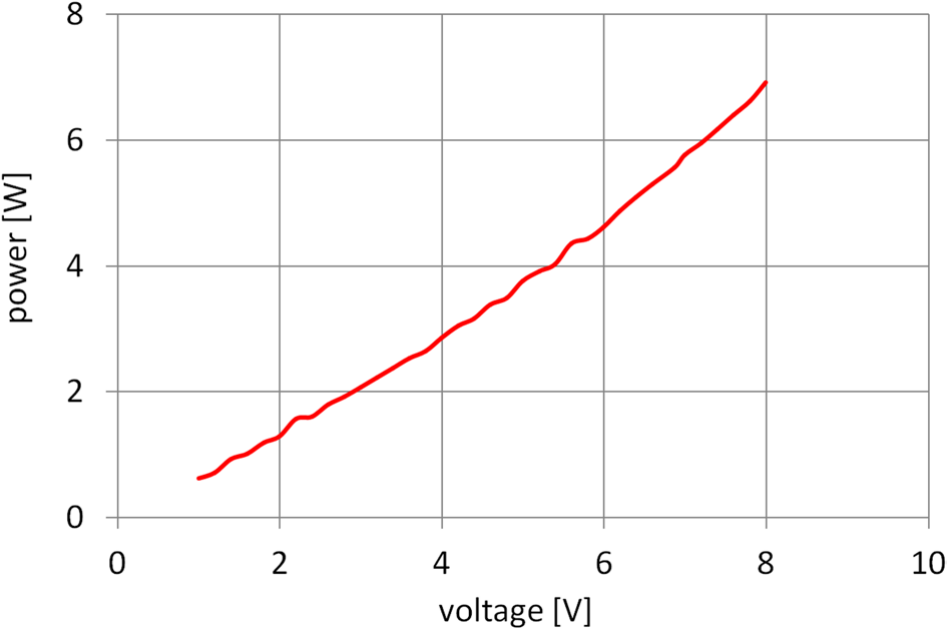
Power losses in the gearset operating at an ambient temperature.

Based on the averaged results of the consecutive measurement steps, the graph shown in Fig. 10 was plotted to show how power consumption by the entire transmission changed as a function of the voltage supplied to the motor. The data could then be used as a basis for further computations and analyses.

In addition, it was established during the experiments that 1-h operation of the gearset resulted in an increase of the temperature inside it by about 1 °C. Hence, it was concluded that the impact of the power lost in the examined drive mechanism on the change in the temperature of the gearset was negligible and could be disregarded. Certainly, as far as heat balance analysis is concerned, that is a simplification. Nevertheless, due to the fact that the insulation of the chamber was not perfect and because of very high thermal capacity of the transmission, the influence of heat sources such as electric phenomena in the running electric motor and energy dissipation processes in the bearings and meshing planetary gears could be ignored without significant consequences for the nature of the conclusions to be drawn.

**Figure 11:**
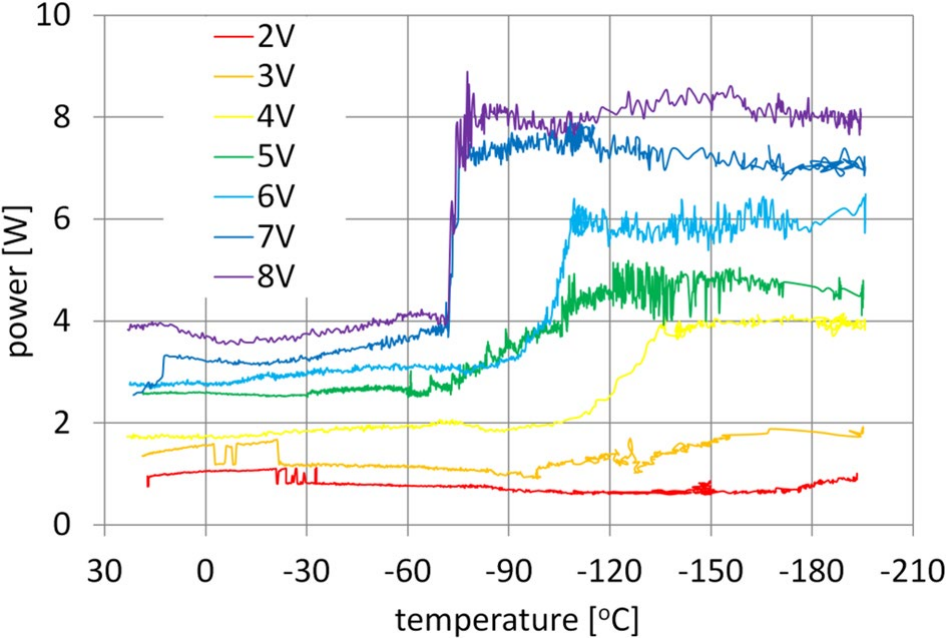
Measurements of the motor power losses.

Measurements began with testing the transmission drive motor. The purpose of this was to determine the power losses of the motor as a function of temperature, so that this value could be included in the analysis of the total power losses of the transmission in the future. This was achieved by cooling the drive motor itself, not connected to the transmission, several times to a temperature below − 190 °C, while supplying it with a voltage of 2 V to 8 V in 1 V steps. Below that range of supply voltage, the engine torque is too low for the test to be carried out over the full temperature range. In this case, after lowering the test temperature below − 70 °C, the engine stopped without any noticeable power increase effect. The full data from the tests are shown in Fig. 11. The graphs clearly show the jump in power consumed by the motor at progressively higher supply voltages, and it was also accompanied by a significant increase in the noise level generated by the motor. This acoustic effect, due to the change in engine operating conditions at low temperature, is probably due to the adverse effect of low temperature on the rotor bearings marked R-2, which, because of their small dimensions, amounting to 1/8 × 3/8 × 5/32 inch, reacted very strongly to large changes in operating temperature. In addition, these elements, due to the highest rotational speed of all the bearings of the mechanism, had a key impact on the total power losses of the entire transmission.

In the next step of the study, an attempt was made to cool the gearset gradually down to temperatures below − 190 °C. The prototype nature of the mechanism mandated that utmost caution be taken while the mechanism was undergoing cooling. For this reason the first test was conducted with the gearset motor supplied with 2 V, which based on preliminary tests was determined to be the lowest voltage at which the motor operated in a fully stable manner. That way, the gearset would be maximally protected against damage caused by the freezing of the gearset parts.

Unfortunately, the 2 V voltage proved to be insufficient and at a temperature of approximately − 82 °C, the increasing resistance in the transmission caused the motor to stall. A similar outcome was observed when the motor was supplied with 3 V. That voltage allowed the transmission to operate to a temperature of − 121 °C, at which the motor shut off. Only when the voltage of the gearset motor had been increased to 4 V could a complete measurement process be carried out, which was concluded when the temperature inside the gearing had dropped below − 190 °C.

During the tests around − 110 °C all Hall sensors reading the engine rotational speed stopped working. This is why data containing this information was not included in the article. Electromechanical motor speed monitoring will be added to the next version of the test bench.

## Analysis of the results

**Figure 12:**
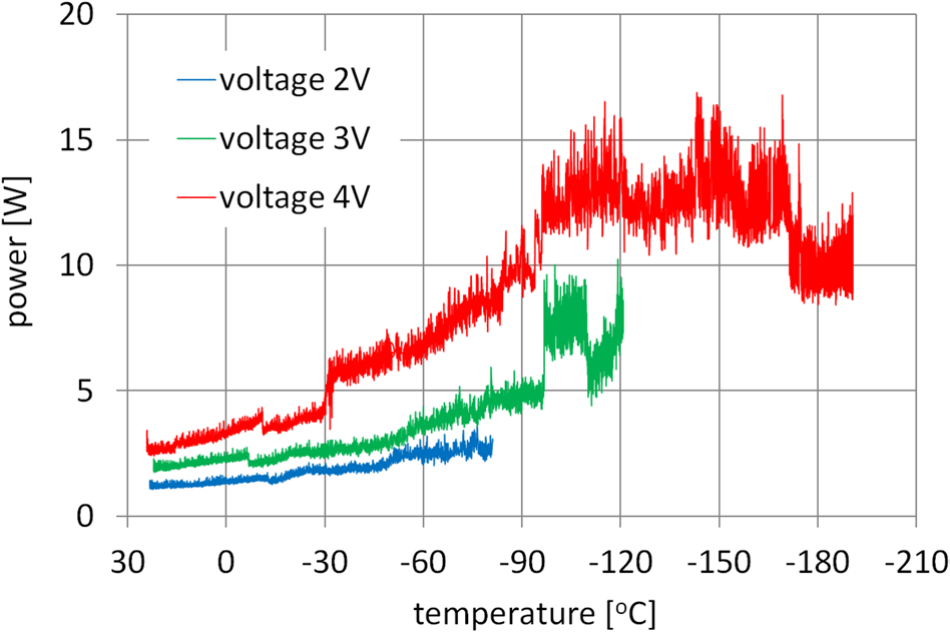
Initial measurements of the gearset power losses.

Supplying the transmission motor with an increasingly higher voltage of the input current causes the speed of the transmission to accelerate proportionally to the value of the supplied voltage. Consequently, the power losses of the gearset also increases proportionally. As the motor supply voltage increases, the demand for power also rises proportionally to the square of the supply voltage. That phenomenon is clearly evident in the graphs presented in Fig. 12, depicting the relation between the transmission power losses and temperature.

There is a gradual increase in the power required to drive the motor regardless of the supply voltage. A spike in the power consumed by the motor, appearing at about − 95 °C, was most likely caused by residual lubricant in the gearbox bearings despite measures taken to remove it. Similar power spikes in the range of − 100 °C to − 110 °C were also observed during the tests of rolling bearing resistance^9^, with the discrepancy in temperatures being a result of a different positioning of the temperature sensor in the gearbox and in the rolling bearings during the tests.

**Figure 13:**
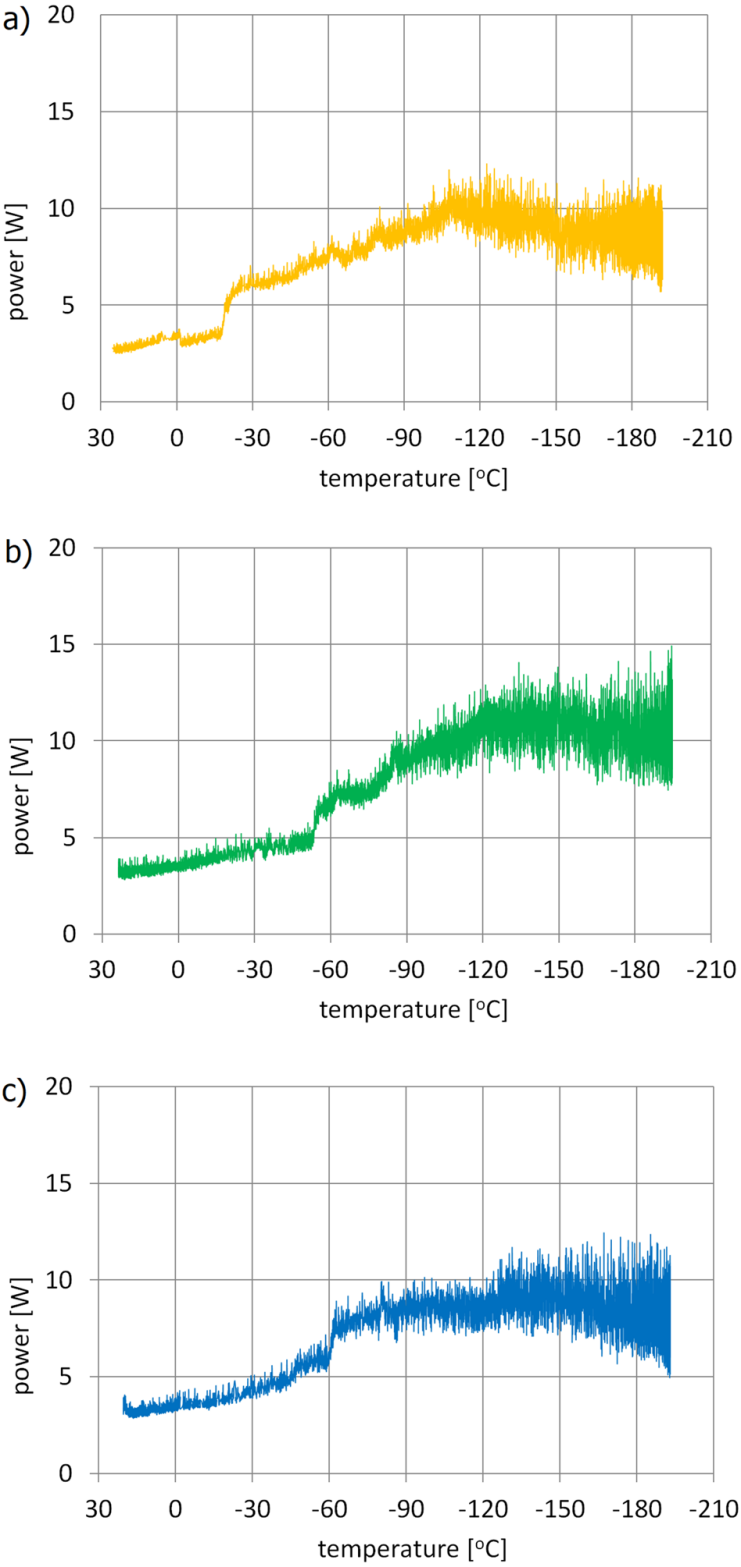
Measurements of the gearset power losses.

To confirm the results, the motor supply voltage was left at 4 V, and the measurements were repeated three more times. Graphs of the full data obtained during these tests are shown in Fig. 13a–c. The number of the data sets collected during the measurements ranged from 6125 to 10,170. The variation can be attributed to the fact that with manual control of the amount of liquid nitrogen supplied to the chamber, it was impossible to maintain identical conditions of the gearbox cooling rate, so the timing of the individual measurements differed slightly. The total cooling time of the gearing during the experiments ranged from 70 to 105 min.

All the measurements demonstrate a similar incremental increase in the power consumed by the motor as a function of the decreasing temperature recorded inside the gearing.

**Figure 14:**
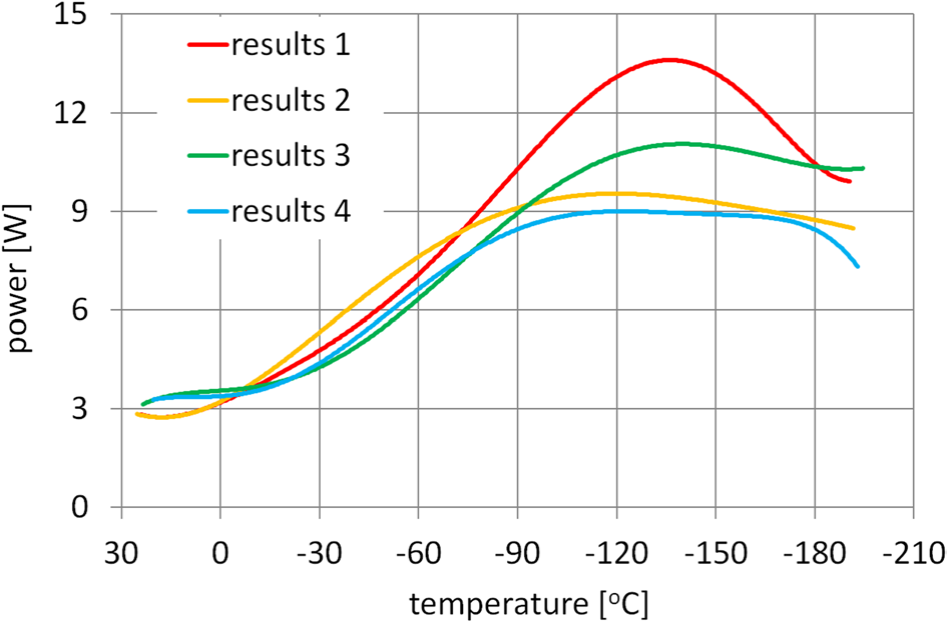
Trend lines of the measurements of the gearbox power losses.

Due to the very large volume of data from each measurement cycle, Fig. 14 shows the polynomial trends for the results from the successive measurements. The coefficients of determination R^2^ for the presented runs vary from 0.8969 to 0.9546. Using the polynomial functions to describe the variation in the power losses as a function of temperature is not intended to accurately reflect the nature of the dependence of the physical phenomena occurring during the motion, but merely to enable an assessment of the variation trend observed during the experiment.

The presented trend lines also facilitate comparison of individual measurement cycles and reveal gradually easing increases in the power required to drive the gearbox during subsequent runs. The phenomenon is most probably caused by the progressive lapping of the meshing elements of the gears and the purging of the ball paths in the bearing raceways of the residue of the original liquid lubricant. A similar phenomenon was also observed during earlier tests of the bearings themselves, described in the article^9^.

**Figure 15:**
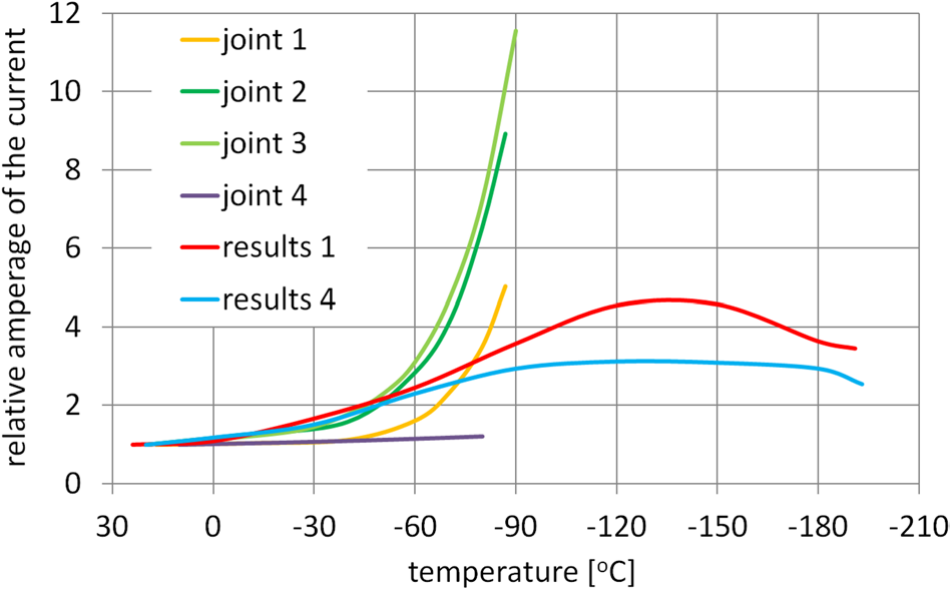
Comparison of the relative amperage of the current supplied to the RA joints of the MVACS lander^8^ and the designed gearset.

The performed experimental tests have demonstrated a gradual increase in the power losses during the operation of the gearbox at progressively lower temperatures. For the examined gearset, the relative increase of the power losses (Fig. 15) once the conditions of the meshing of the gearset components had stabilized was approximately 300% in the temperature range adopted in the study. In terms of absolute values, the power required to overcome the resistance was easily achieved by the motor deployed in the transmission drive system across the entire temperature range. The increase in the power losses of the gears under laboratory conditions had a much gentler course than it has been reported in the literature. It was referred to the relations observed under laboratory conditions describing the impact of temperature on the amperage of the current consumed by the motors of the actuators of the robotic arm of the Mars Volatiles and Climate Surveyor lander. For the drive mechanisms of this lander the relative increase in the amperage of the current supplied to the motors during their operation at increasingly lower temperatures ranged from 120 to 1150%, depending on the joint. Furthermore, the lander mechanical systems were designed to operate at temperatures not lower than − 105 °C (− 90 °C), and below that temperature they would be shut down to avoid damage.

Figure 15 shows a comparison of the relative amperage of the current supplied to the RA joints of the MVACS lander^8^ and the designed gearbox. The measurement points connected by the lines labeled as Joint 1–4 were produced by dividing the value of the current amperage read for each measurement point by the value of the current amperage at an ambient temperature. The curves marked as measurement 1 and 4 show the trend lines obtained from the first and last measurement cycles of the amperage of the current supplied to the motor of the investigated gearset. Relative values were obtained by dividing the values of the amperage of the current for each measurement point by the value of the amperage of the current at an ambient temperature. As a result of those procedures the presented values are dimensionless, which facilitates their comparison.

The analysis of these results leads to the conclusion that the value of relative amperage of the current powering the drive motors of the mechanical systems of joints 1–3 of the RA joints of the MVACS lander^8^ increases considerably more during operation at lower temperatures than in the case of the designed gearbox. That does not apply to joint 4 driven by a very small gearset generating a maximum torque of only 10 Nm and tested only at temperatures equal to or higher than − 80 °C. Also, the operating temperature limit of − 190 °C achieved for the tested gearbox is much lower than the limit of − 80 °C to − 90 °C for the RA mechanical systems. Thus, the transmission presented in this paper is capable of operating at a much lower temperature than the RA joints of the MVACS lander. Its performance tests under load will allow researchers to define its technical capacity and determine possible time of operation under low-temperature conditions.

## Conclusions

Multiple tests of the gearbox cooled down to temperatures below − 190 °C have shown that gearing built of elements made from materials with different coefficients of thermal expansion may operate under such conditions. The necessary conditions for effective performance of the mechanism at very low temperatures are properly designed and experimentally tested elements of the gearing as well as structural nodes that enable connection and interaction of elements with different coefficients of thermal expansion.

Application of modern, lightweight structural materials under ultra-low temperature conditions paves the way for the construction of lighter structures capable of overcoming the constraints inherent in the conditions prevailing in outer space and on other planets, particularly on Mars.

## Data Availability

The datasets used and/or analyzed during the current study available from the corresponding author on reasonable request.
